# *Candida auris* in a U.S. Patient with Carbapenemase-Producing Organisms and Recent Hospitalization in Kenya

**DOI:** 10.15585/mmwr.mm6830a3

**Published:** 2019-08-02

**Authors:** Richard B. Brooks, Maroya Walters, Kaitlin Forsberg, Elisabeth Vaeth, Kate Woodworth, Snigdha Vallabhaneni

**Affiliations:** ^1^Division of Healthcare Quality Promotion, National Center for Emerging and Zoonotic Infectious Diseases, CDC; ^2^Infectious Disease Epidemiology and Outbreak Response Bureau, Maryland Department of Health; ^3^Division of Foodborne, Waterborne, and Environmental Diseases, National Center for Emerging and Zoonotic Infectious Diseases, CDC; ^4^IHRC, Inc., Atlanta, GA.

*Candida auris* is an emerging drug-resistant yeast that causes outbreaks in health care facilities; cases have been reported from approximately 30 countries. U.S. cases of *C. auris* are likely the result of importation from abroad followed by extensive local transmission in health care settings (*1*). Early detection of *Candida auris* is key to preventing its spread. *C. auris* frequently co-occurs with carbapenemase-producing organisms (CPOs), like carbapenem-resistant Enterobacteriaceae (CRE), organisms for which testing and public health response capacity substantially increased beginning in 2017. In September 2018, the Maryland Department of Health (MDH) was notified of a hospitalized resident with CPO infection and colonization and recent hospitalization in Kenya. In light of this history, the patient was screened for *C. auris* and found to be colonized. Public health responses to CPOs can aid in the early identification of *C. auris*. As part of CPO investigations, health departments should assess whether the patient has risk factors for *C. auris* and ensure that patients at risk are tested promptly.

First identified in Japan in 2009, *C. auris* is an emerging drug-resistant yeast that has now been reported in approximately 30 countries (*2*). *C. auris* has been associated with outbreaks in health care facilities, where its spread is facilitated by challenges with identification, persistent contamination of the health care environment, and limited effectiveness of some standard hospital disinfectants. In the United States, outbreaks have most frequently occurred in high-acuity postacute care facilities, including nursing homes that care for mechanically ventilated patients. Co-infection or co-colonization with *C. auris* and other emerging multidrug-resistant organisms, including CPOs, has been observed regularly.

In September 2018, the MDH was notified about a patient who had recently been medically evacuated from Kenya to an acute care hospital in Maryland. The patient was a U.S. resident who did not work in health care and who had a cerebral hemorrhage while visiting Kenya. During the subsequent month-long hospitalization in Kenya, the patient underwent several operations and other procedures, including arterial clipping and placement of a tracheostomy and feeding tube. Hospital treatment was complicated by sepsis, pneumonia, and a urinary tract infection, requiring treatment with broad-spectrum antibiotics and at least one course of antifungal medications.

In light of the patient’s history of receiving health care abroad, the Maryland hospital placed the patient on contact precautions in a private room immediately upon admission (*3*). Specimens collected at admission to evaluate ongoing fevers grew several highly drug-resistant organisms, including oxacillinase-48-like-producing carbapenem-resistant *Klebsiella pneumoniae* in urine and New Delhi metallo-beta-lactamase-producing carbapenem-resistant *Pseudomonas aeruginosa* in sputum.

At the time of the investigation, *C.* *auris* had been reported from one major hospital in Kenya, although not from the facility where the Maryland patient had been hospitalized (*4*). MDH had previously identified *C. auris* colonization in a patient infected with multiple CPOs and who had had a recent prolonged hospitalization in India. Based on the current patient’s prolonged hospitalization in a country with known *C. auris* cases, the patient’s colonization and infection with CPOs, and MDH’s previous experience, MDH, in consultation with CDC, recommended that the hospital evaluate the patient for *C. auris* colonization. On hospital day 12, a single skin swab of the patient’s bilateral axilla and groin areas (one swab for all four areas) was obtained for fungal culture; resulting growth was identified as *C. auris* by matrix-assisted laser desorption/ionization time-of-flight mass spectrometry, indicating colonization in the absence of clinical signs and symptoms. Consistent with *C. auris* detection representing colonization rather than infection, the patient did not receive antifungal therapy while hospitalized in the United States and was ultimately discharged to a rehabilitation facility. Because of the potential for *C. auris* to be transmitted in health care settings (*5*), 21 patients located on the same hospital unit as the index patient were evaluated for *C. auris* colonization. All screening swabs were negative for *C. auris*.

## Discussion

*C. auris* colonization was identified in a hospitalized patient with a recent history of hospitalization in Kenya and CPO infection and colonization. Transmission of *C. auris* and CPOs to other patients was likely prevented because of the hospital’s rapid recognition of the patient’s high risk for multidrug-resistant organism colonization and immediate use of appropriate contact precautions upon admission. In facilities where patients with *C. auris* have not been immediately identified, and specific infection control measures were not implemented, transmission to other patients has occurred: in one long-term care facility ventilator unit, nearly half of patients became colonized with *C. auris* within months of the index patient’s admission to the facility (*6*). This case highlights the importance of a high level of suspicion for *C. auris* in persons admitted to U.S. health care facilities with a history of health care abroad, even if *C. auris* is not known to be widespread in that location. Early identification of *C. auris* is critical to preventing further transmission.

To date, 11 other patients with *C. auris* infection or colonization have been identified in the United States who had a recent history of hospitalization abroad, including in India, Pakistan, South Africa, the United Arab Emirates, and Venezuela. At least six of the 11 patients were also colonized with CPOs; co-colonization might have been higher because not all patients were assessed for CPO colonization. Whole genome sequencing demonstrated that the *C. auris* isolates from these 12 patients, including the patient described in this report, were in the same clades as isolates from the countries where the patients received health care (*1*).

CDC recommends screening for *C. auris* colonization for patients who have had an overnight stay in a health care facility outside the United States in the preceding 12 months, especially if care occurred in a country with documented *C. auris* infections (*7*). This is in addition to the 2013 CDC recommendation that facilities place patients who have had overnight stays in health care facilities outside the United States within the past 6 months on contact precautions and perform screening for CPOs like CRE (*8*). Health care facilities should develop strategies to consistently and reliably obtain patients’ travel histories for medical care received outside of the United States in order to identify patients to be screened, and patients should inform their health care providers about any health care received abroad to inform their care (*3*).

As exemplified by this episode and other *C. auris* outbreak investigations, co-colonization with *C. auris* and CPOs is common in critically ill patients (50% of patients with *C. auris* are also colonized with a CPO) (*9*). CPO detection capacity has increased in the United States since 2017, and CDC recommends a public health response to even single cases of unusual resistance, including most CPOs (*10*). The public health investigation of CPOs should include an assessment of whether the patient had overnight health care exposures in countries where *C. auris* has been identified; patients not previously screened for *C. auris* should be promptly tested. In addition, if yeast is identified on any clinical cultures in such patients, it should be identified to the species level regardless of body site source. Confirmatory testing for *C. auris*, carbapenemase testing for Enterobacteriaceae, *Pseudomonas aeruginosa*, and *Acinetobacter baumannii*, and colonization screening for CPOs and *C. auris* is available free of charge through the Antibiotic Resistance Laboratory Network.* Globally, it is critical to prevent the emergence and spread of highly drug-resistant organisms like *C. auris* and CPOs. Public health investigations of CPOs could facilitate early detection of *C. auris* and might lead to earlier detection of this organism, thus preventing its spread.

**Summary**

**What is already known about this topic?**

*Candida auris* is an emerging drug-resistant yeast of high public health concern.

**What is added by this report?**

A Maryland resident with hospitalization in Kenya and carbapenemase-producing organism (CPO) colonization/infection was screened and found to be colonized with *C. auris,* demonstrating that CPO investigations can facilitate early identification of *C. auris*.

**What are the implications for public health practice?**

Health care exposure outside the United States and CPO colonization/infection are *C. auris* risk factors. CPO case investigations can provide opportunities to identify patients with overnight hospitalization outside the United States during the previous year, enabling early detection of *C. auris* if CDC recommendations to screen such patients for *C. auris* colonization are followed.

## Footnotes

* https://www.cdc.gov/drugresistance/solutions-initiative/ar-lab-network.html.
